# Research on Knowledge, Attitudes, and Practices of Influenza Vaccination Among Healthcare Workers in Chongqing, China—Based on Structural Equation Model

**DOI:** 10.3389/fpubh.2022.853041

**Published:** 2022-05-19

**Authors:** Siyu Chen, Yueming Jiang, Xiaojun Tang, Lin Gan, Yu Xiong, Tao Chen, Bin Peng

**Affiliations:** ^1^Department of Epidemiology and Health Statistics, School of Public Health and Management, Chongqing Medical University, Chongqing, China; ^2^Clinical 5+3 Integration, The Second Affiliated Hospital of Chongqing Medical University, Chongqing, China; ^3^Chongqing Center for Disease Control and Prevention, Chongqing, China; ^4^National Influenza Center, Institute of Viral Disease Control and Prevention, Chinese Center for Disease Control and Prevention, World Health Organization Influenza Reference and Research Cooperation Center, Beijing, China

**Keywords:** knowledge, attitudes, practices, influenza vaccination, health care workers, structural equation model

## Abstract

**Background:**

Influenza is associated with a large number of disease burdens, and it is generally recommended that all healthcare workers (HCWs) get an influenza vaccination. However, the vaccination rates among HCWs are still low. This study aimed to assess HCWs' knowledge, attitude, and practice (KAP) about the influenza vaccine, and by establishing a structural equation model (SEM) to explore the influencing factors of medical personnel's influenza vaccination in Chongqing, China.

**Methods:**

From September to November 2019, we conducted a cross-sectional survey in four sentinel hospitals and four non-sentinel hospitals in Chongqing, China. We calculated knowledge, attitude, and behavior scores for each study participant and assessed the level of knowledge, attitude, and behavior of the medical staff. An SEM was used to evaluate the relationship between latent variables, and the path graph between knowledge, attitude, and behavior was established.

**Results:**

A total of 1,412 valid questionnaires were collected in this survey, including four sentinel hospitals (*N* = 606, 42.92%) and four non-sentinel hospitals (*N* = 806, 57.08%). Women (*N* = 1,102, 78.05%) were more than men (*N* = 310, 21.95%), with an average age of 32.36 ± 7.78 years old and under 30 years old (*N* = 737, 52.20%), respectively. Nurses (741, 52.48%) were the main subjects, followed by physicians (457, 32.37%). The final SEM model was obtained after the model was modified and adjusted. A bootstrap analysis of path coefficients was carried out on the final model. Knowledge has a direct influence on behavior. The normalized path coefficient is 0.071 (95% CI: 0.002–0.161), and the value of *P* of the hypothesis test result of the path coefficient is 0.042. The direct influence of knowledge on attitude standardization was 0.175 (95% CI: 0.095–0.281). The direct influence of attitude on practice standardization was 0.818 (95% CI: 0.770–0.862). The indirect effect of knowledge on the standardization of practice through attitude was 0.144 (95% CI: 0.076–0.235).

**Conclusions:**

According to the SEM, there is a direct positive correlation between KAP and the influenza vaccine. The indirect influence of knowledge on the standard of behavior through attitude is about two times as much as the direct influence on behavior, indicating that attitude plays a strong mediating role between knowledge and practice.

## Introduction

Influenza is an acute respiratory infectious disease caused by influenza viruses that can lead to serious repercussions on the health of an individual. The influenza virus is highly antigenic and spreads rapidly. It can cause seasonal epidemics every year, and outbreaks can occur in places where people gather, such as hospitals, schools, and nursing homes. Annual seasonal influenza epidemics have a significant impact on the global population in terms of morbidity and mortality; there are an estimated one billion influenza cases each year, of which 3–5 million are severe cases, leading to 290,000–650,000 influenza-related respiratory deaths (1). In particular, in the midst of the coronavirus disease 2019 (COVID-19) global pandemic, there may be a risk of COVID-19 combined with influenza and other respiratory infections this winter and next spring. Vaccination against influenza is the key to reduce the incidence rate of influenza and its related social and economic burden (2). Even if the national influenza virus vaccination guidelines are different, it is generally recommended that we vaccinate not only high-risk patients with pre-existing or high exposure risk, but also the whole population (6 months or more), which decreases the risk of personal infection and improves the immunity of the population (3).

In recent years, there are few studies on the health burden of influenza among healthcare workers (HCWs), especially in domestic data. Previous studies have found that compared with the general population, medical staff have more contact with influenza patients, so the risk of infection with influenza viruses is higher than that of the general population (4). A meta-analysis of 29 global studies showed that the average laboratory-confirmed incidence of influenza per season among unvaccinated medical personnel was about 18.7% (95% CI: 15.8–22.1%), which was 3.4 (95% CI: 1.2–5.7) times higher than among healthy adults (5). A systematic review published in 2016 showed that during the Influenza A (H1N1) pandemic, healthcare professionals were at a higher risk of infection than the general population (odds ratio [OR] = 2.08, 95% CI: 1.73–2.51), while clinicians with direct patient interaction were at a higher risk (OR = 6.03, 95% CI: 2.11–17.8) (6). The World Health Organization (WHO) conducted a rapid assessment of evidence in 2019 also suggested that HCWs were at a higher risk of influenza virus infection than the general population (7), and that influenza virus infection among HCWs may increase the risk of nosocomial infection.

The knowledge, attitude, and practice (KAP) theory model emphasizes the importance of knowledge and attitude in behavioral decision-making, explains the generation of health behavior and predicts the change of behavior by exploring the KAP of high-risk groups (8). KAP theory holds that health knowledge is the basis for establishing a positive attitude and healthy behavior, and attitude is the driving force for behavior change, and the goal is to promote healthy behavior (9).

We know from KAP theory that there is a causal relationship between KAP (10). However, KAP are potential variables that are difficult to measure directly. A structural equation model (SEM) is a new multivariate statistical technology that integrates the traditional statistical analysis methods, such as confirmatory factor analysis, path analysis, and multiple regression analysis. It can deal with potential variables and observe indicators and measurement errors (11). In addition, it can also explore the causal relationship between potential variables and quantitatively evaluate the direct and indirect effects of variables (12).

Variables are divided into explicit variables and potential variables. Potential variables have characteristics that cannot be directly measured, such as knowledge, attitude, and behavior variables in the KAP mode (13). Although knowledge, attitude, and behavior cannot be measured directly, information about the research object can be obtained through a questionnaire. Therefore, we used a questionnaire, which is also a common method in KAP research.

This study aimed to evaluate the associations among KAP regarding the influenza vaccine among HCWs in Chongqing, China based on the KAP theory using an SEM approach.

At present, there is some research on the theory of KAP of HCKs regarding vaccinations in China, but there are few types of research on the relationship among KAP by using the SEM. Because of the rare data, a study needs to target the local Chinese HCWs' KAP for determination of the influenza vaccination status and impact among them. As far as we are concerned, this is the first study that aims to establish the structural equation modeling to explore the relationship among the HCWs' KAP about influenza vaccination in Chongqing, China.

## Method

### Study Subjects

We used the multistage sampling technique to conduct this cross-sectional survey.

In the first stage, hospitals were divided into sentinel hospitals and non-sentinel hospitals according to whether they participated in the National Influenza Surveillance System. We randomly selected four out of eight sentinel hospitals in Chongqing, and randomly selected four non-sentinel hospitals where the selected sentinel hospitals were located as the research site. In the second stage, departments were divided into high-risk and low-risk departments according to whether they had usual contact with influenza patients. HCWs in high-risk departments, such as respiratory, infection, emergency, pediatrics, and fever clinic department, were randomly selected in each selected hospital as the study subjects. The same in low-risk departments, such as general surgery, obstetrics and gynecology, laboratory, and radiology, were randomly selected in each selected hospital as the study subjects. All participants who had worked in the hospitals for at least 1 year and gave their written and informed consent were eligible for inclusion.

### Study Design and Data Collection

A cross-sectional questionnaire survey was conducted by us from September to November 2019. Before the formal investigation, pilot tests were conducted on 50 HCWs to assess accessibility and comprehension, and the questionnaire was revised based on received feedback. Questionnaires are distributed online and offline; respondents can choose to complete paper questionnaires on-site or submit electronic questionnaires through the questionnaire survey website. (Chinese popular online survey platform: http://www.wjx.cn). Before the start of each face-to-face survey, an investigator went to the office, explained the research to the participating HCWs, and required them for their consent to participate in the research. Participants were also required to sign an informed consent form before filling out the electronic questionnaire.

Influenza vaccine-rated knowledge consists of eight items, for example, “Influenza is transmitted primarily by coughing and sneezing.” (K1); “The influenza shot contains live viruses but cannot cause people to get influenza.” (K2); “The best time for influenza vaccination is before the influenza season.” (K3); “The side effects of the influenza vaccine include headaches” (K4); “The most recommended groups for influenza vaccination include frail people particularly who suffer from chronic diseases”(K5); “The most recommended groups for influenza vaccination include HCWs, pupils, kindergarten children, and pregnant women” (K6–K8). Response options were “Agree” or “Disagree or do not know.” The correct answer (agree) was scored 1 and the incorrect answer (disagree or do not know) was scored 0. The final scores of influenza vaccine-rated knowledge ranged from 0 to 8. Higher scores indicated better influenza vaccine knowledge. The total awareness rate of influenza vaccine knowledge was equal to the total number of knowledge questions answered correctly/(the number of knowledge items in each questionnaire × the number of effective response participants) × 100%. The awareness rate of each influenza vaccine knowledge question was equal to the number of participants answered correctly/the number of effective response participants × 100%.

There were four statements set in the attitude section to assess the attitude of HCWs toward the influenza vaccine. They included “I think it's necessary to get the influenza vaccine.”(A1); “I don't worry about the side effects of the influenza vaccine.”(A2); “I think even if I never get influenza, I still need to vaccinate the influenza vaccine.”(A3); “It's important for me to get the influenza vaccine every year.” (A4). A five-point Likert scale was used to record the response of the participants, such as “strongly agree,” “agree,” “neutral,” “disagree,” and “strongly disagree” in each question. Responses that included “strongly agree” and “agree” were considered to agree with or have a positive attitude toward the statement, while the other responses were considered disagreement or having a negative attitude. To determine the attitude score, we assigned 0–4 points from “strongly disagree” to “strongly agree” respectively to each item, and the total attitude score ranged from 0 to 16. Higher scores represented more positive attitudes toward the influenza vaccine. The overall retention rate of a positive attitude toward the influenza vaccine was equal to the total number of positive attitudes questions/(the number of attitudes items in each questionnaire × the number of effective response participants × 100%). The holding rate of each positive attitudes question was equal to the number of participants who opted “Agree”/the number of effective response participants × 100%.

Influenza vaccine-rated practice was considered using five items, which include “Have you received the influenza vaccine in the past year?”(P1); “Do you take the initiative to learn about flu vaccine-related information?”(P2); “Did you recommend the flu vaccine to the patient in the past year?”(P3); “Are you willing to get the flu vaccination this year?”(P4); “Did you want your family members to get the flu vaccine?”(P5). Participants will obtain 1 score when they answered the question “Yes” and get no score if they say “No”, except for question P4. A five-point Likert scale was used to assess the response of the question P4, such as “strongly agree,” “agree,” “neutral,” “disagree,” and participants get 0–4 points from “strongly disagree” to “strongly agree.” The total practice score ranged from 0 to 8.

### Statistical Analysis

For data analysis, IBM^®^ SPSS^®^ Statistics 26.0 and IBM^®^ SPSS^®^ Amos™ 24.0 were used.

Mean ± standard deviation (SD) or frequency and percentage is used to describe demographic information. We used Spearman's theory to assess the correlation between latent variables. All differences were evaluated using two-tailed tests, and the significance level was set at *P* < 0.05.

An SEM was constructed to determine the relationship between influenza vaccine KAP.

The maximum likelihood estimate (MLE) was used for parameter estimation, and the test level was set to α = 0.05. We used the chi-square/degrees of freedom (CMDN/DF), root mean square error of approximation (RMSEA), the goodness of fit index (GFI), adjusted goodness of fit index (AGFI), normed fit index (NFI), incremental fit index (IFI), comparative fit index (CFI), parsimonious goodness of fit index (PGFI), and other indicators to evaluate the fitting effect of the model. A value of CMDN/DF < 3.00, RMSEA < 0.05, GFI > 0.90, AGFI > 0.90, NFI > 0.90, IFI > 0.90, CFI > 0.90, and PGFI > 0.50 can support a good model fit (14).

The bootstrap method was used to test the significance of the mediating effect of related variables in the ideal model. In addition, a bias-corrected bootstrap 95% CI was used to examine the significance of direct and indirect effects (15).

**Figure 1:**
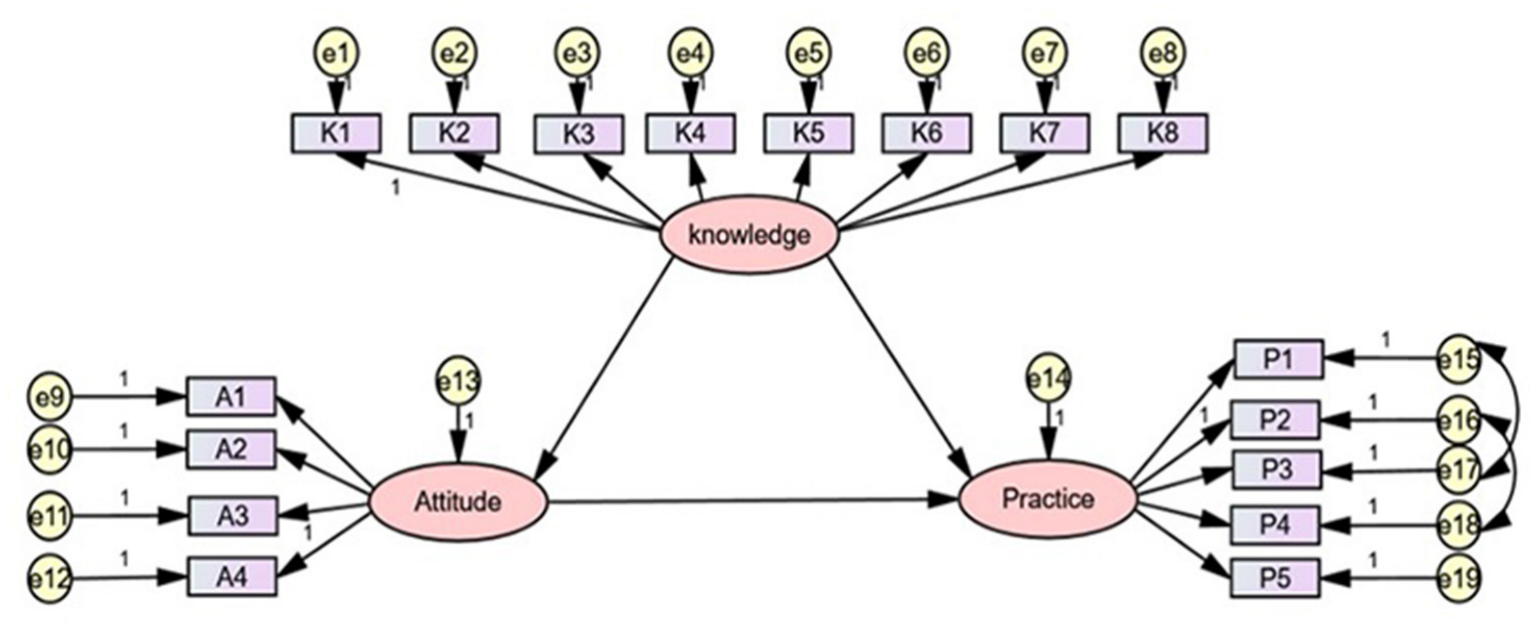
The ideal SEM. Rectangle shows observed variables, ellipses indicate potential variables, and circles represent residual terms.

According to our hypothesis, the ideal SEM was established, which was about the association among influenza vaccine-rated KAP in a sample of HCWs in Chongqing, China. We removed some corresponding paths, because the path coefficients of “knowledge” on “attitudes,” and “attitudes” on “practice” were not statistically significant in the ideal SEM fitting results (All *P* > 0.05). Ideal SEM is shown in Figure 1.

The Cronbach's α value of the final SEM was 0.705 and the Kaiser-Meyer-Olkin (KMO) value was 0.817, showing good reliability and validity (16).

## Results

### Sample Characteristic

This survey collected 1,412 valid questionnaires, covering four sentinel hospitals (*N* = 606, 42.92%) and four non-sentinel hospitals (*N* = 806, 57.08%).There are more women (*N* = 1,102, 78.05%) than men (*N* = 310, 21.95%), the mean age is 32.36 ± 7.78 years, and most of the participants were 30 years old or younger (*N* = 737, 52.20%). The respondents were mainly nurses (*N* = 741, 52.48%), followed by physicians (*N* = 457, 32.37%), medical technicians, and others (*N* = 214, 15.16%). The details of demographic characteristics are shown in Table 1.

**Table 1:** Demographic characteristics of 1,412 healthcare workers (HCWs) with influenza vaccination or not.

**Variable**	**Total (%)**	**Vaccinated**	**Unvaccinated**
Type of hospital
Non-sentinel hospital	806 (57.08)	117 (14.52)	689 (85.48)
Sentinel hospital	606 (42.92)	120 (19.80)	486 (80.20)
Sex
Males	310 (21.95)	43 (13.87)	267 (86.13)
Females	1,102 (78.05)	194 (17.60)	908 (82.40)
Age
≤ 30	737 (52.20)	108 (14.65)	629 (85.35)
30~	466 (33.00)	88 (18.88)	378 (81.12)
40~	161 (11.40)	29 (18.01)	132 (81.99)
50~	48 (3.40)	12 (25.00)	36 (75.00)
Educational attainment
College degree and below	310 (21.95)	47 (15.16)	263 (84.84)
Bachelor degree	908 (64.31)	158 (17.40)	750 (82.60)
Postgraduate and above	194 (13.74)	32 (16.49)	162 (83.51)
Marital status
Married	372 (26.35)	170 (17.03)	828 (82.97)
Unmarried	998 (70.68)	58 (15.59)	314 (84.41)
Others	42 (2.97)	9 (21.43)	33 (78.57)
Department
Low-risk	784 (55.52)	95 (12.12)	689 (87.88)
High-risk	628 (44.48)	142 (22.61)	486 (77.39)
Profession
Physician	457 (32.37)	79 (17.29)	378 (82.71)
Nurse	741 (52.48)	136 (18.35)	605 (81.65)
Others	214 (15.16)	22 (10.28)	192 (89.72)
Years in profession
≤ 5	536 (37.96)	68 (12.69)	468 (87.31)
6 10	441 (31.23)	80 (18.14)	361 (81.86)
11~15	202 (14.31)	43 (21.29)	159 (78.71)
≥16	233 (16.50)	46 (19.74)	187 (80.26)
Professional titles
Primary	891 (63.10)	138 (15.49)	753 (84.51)
Junior	394 (27.90)	75 (19.04)	319 (80.96)
Senior	127 (8.99)	24 (18.90)	103 (81.10)

### Descriptive Analysis for Influenza Vaccine-Related KAP

The overall awareness rate of influenza vaccine knowledge among HCWs was 82.83%. The overall retention rate of positive attitudes toward the influenza vaccine was 63.07%, which was lower than that of knowledge. The total execution rate of right practice toward influenza vaccine among HCWs was 47.29%. The detailed values are listed in Table 2.

**Table 2:** Descriptive statistics for influenza vaccine-related knowledge, attitudes, and practice (KAP).

	***M* ± SD (range)**	***N* (%)**		***M* ± SD (range)**	***N* (%)**		***M* ± SD (range)**	***N* (%)**
K1	0.99 ± 0.12 (0–1)	1,393 (98.65)	A1	3.12 ± 0.66 (0–4)	1,214 (85.98)	P1	0.17 ± 0.37 (0–1)	237 (16.78)
K2	0.86 ± 0.35 (0–1)	1,211 (85.76)	A2	2.12 ± 0.86 (0–4)	500 (35.41)	P2	0.69 ± 0.46 (0–1)	987 (68.77)
K3	0.73 ± 0.45 (0–1)	1,024 (72.52)	A3	2.94 ± 0.75 (0–4)	1,106 (78.33)	P3	0.18 ± 0.39 (0–1)	259 (18.34)
K4	0.88 ± 0.33 (0–1)	1,237 (87.61)	A4	2.60 ± 0.82 (0–4)	742 (52.55)	P4	2.56 ± 1.03 (0–4)	730 (51.70)
K5	0.90 ± 0.31 (0–1)	1,265 (89.59)				P5	0.81 ± 0.39 (0–1)	1,142 (80.88)
K6	0.93 ± 0.25 (0–1)	1,316 (93.20)						
K7	0.95 ± 0.21 (0–1)	1,347 (95.40)						
K8	0.40 ± 0.49 (0–1)	564 (39.94)						
k	6.63 ± 2.50 (0–8)	82.83%	A	10.78 ± 3.08 (0–16)	63.07%	P	4.41 ± 2.65 (0–8)	47.29%

*M, mean; SD, standard deviation; K, overall awareness rate of influenza vaccine-related knowledge; A, overall retention rate of a positive attitude toward influenza vaccine; P, total execution rate of right practice toward influenza vaccine among HCWs*.

### Correlation Analysis Among Latent Variables

We used Spearman's correlation to analyze the correlation among KAP one by one. There were positive correlations among influenza vaccine-related KAP (*r* = 0.177, 0.217, and 0.855, all the values of *P* were significant). The details are shown in Table 3.

**Table 3:** The correlation coefficient among latent variables.

**Parameter**	**Correlation coefficient**	***P*-value**
Knowledge < ->Attitudes	0.177	0.007
Knowledge < ->Practice	0.217	0.007
Attitudes < ->Practice	0.855	0.016

### Evaluation of the Fitting Effect of the Model

In addition, the covariant relationships between e15 and e17 were established, as well as between e16 and e18. Through repeated modification and fitting of the model, the fit indices of SEM finally reached the adaptation standards: CMDN/DF = 2.068, RMSEA = 0.028, GFI = 0.981, AGFI = 0.974, NFI = 0.923, IFI = 0.959, CFI = 0.958, and PGFI = 0.731. The results are shown in Table 4.

**Table 4:** The fit indices of structural equation model (SEM).

**Fit index**	**Goodness of fit index of SEM**							
	**CMDN/DF**	**RMSEA**	**GFI**	**AGFI**	**NFI**	**IFI**	**CFI**	**PGFI**
Reference index	<3.00	<0.05	>0.90	>0.90	>0.90	>0.90	>0.90	>0.50
Final model index	2.068	0.028	0.981	0.974	0.923	0.959	0.958	0.731

### Structural Equation Modeling

**Figure 2:**
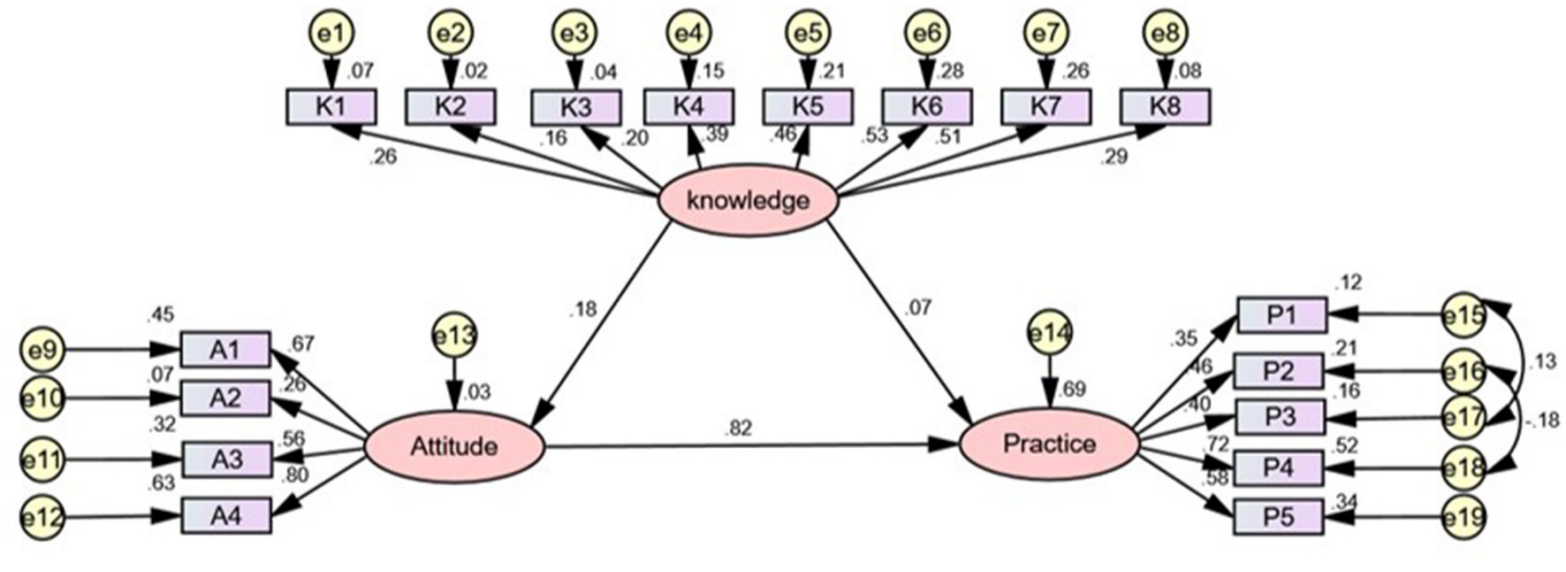
The final SEM. Rectangle shows observed variables, ellipses indicate potential variables, and circles represent residual terms. The values of single-headed arrows represent the standardized coefficients. All paths were significant (*P* < 0.05).

Figure 2 shows the final SEM. Table 5 presented the results of hypothesis testing for trajectory coefficients of knowledge, attitudes, and behavior. Table 6 illustrated the bootstrap analysis of mediating effect significance test for the final model. As is shown in Figure 2 and Tables 5, 6: knowledge had a standardized direct effect on practice, with a value of 0.071, 95% CI: 0.002–0.161, the value of *P* of the hypothesis testing results for path coefficients was 0.042, which was significant; the standardized direct effect of knowledge on attitude was 0.175 with 95% CI: 0.095–0.281; attitude had a standardized direct effect on practice, which was 0.818, 95% CI: 0.770–0.862; the standardized indirect effect of knowledge on practice through attitudes was 0.144, 95% CI: 0.076–0.235. The standardized total effects of knowledge on behavior were 0.215.

**Table 5:** Hypothesis testing results for path coefficients of knowledge, attitude and practice.

**Statements**	**Unstandardized** **estimates**	**Standardized** **estimates**	**SE**	***T*-value**	***P*-value**
Knowledge → attitude	3.850	0.175	1.014	3.794	0.007
Knowledge → practice	0.512	0.071	0.260	1.970	0.042
Attitude → practice	0.268	0.818	0.020	13.177	0.018

**Table 6:** Bootstrap analysis of mediating effect significance test for the final mode.

**Model paths**	**Standardized direct effects**	***P*-value**	**95% CI**		**Standardized indirect effects**	***P*-value**	**95% CI**	
			**LLCI**	**ULCI**			**LLCI**	**ULCI**
Knowledge → attitude	0.175	0.007	0.095	0.281	–	–	–	–
Knowledge → practice	0.071	0.042	0.002	0.161	–	–	–	–
Attitude → practice	0.818	0.018	0.770	0.862	–	–	–	–
Knowledge → practice	–	–	–	–	0.144	0.007	0.076	0.235

## Discussion

Our study aimed to explore the relationship between influenza vaccine-rated knowledge, attitudes, and practice, among HCWs in Chongqing, China.

The vaccine-related knowledge might be an important influential factor for improving influenza vaccination status among HCWs as it may lead to good attitude and practice, which ultimately boost influenza vaccination status. So far, there are few investigations or similar studies about the knowledge, attitudes, and practice on HCWs in our country based on KAP theory.

Previous research showed that the coverage rate of the influenza vaccine was extremely low among medical staff, though the government encourages medical staff to prioritize vaccine. Our study indicated that the overall awareness rate of influenza vaccine-related knowledge was 82.83%, which was satisfactory. This was much higher than the investigation of Austria (66.4%) (17). This research also found that the total retention rate of a positive attitude toward the influenza vaccine was 63.07%, which was significantly higher compared with a similar study (18). However, the final execution rate of right practice toward influenza vaccination among HCWs was 47.29%, which was not optimistic, suggesting that improvement and increased social awareness is needed. In particular, regarding the question P1 “Have you received the influenza vaccine in the past year,” the influenza vaccination coverage of HCWs in Chongqing during the 2018/2019 influenza season, was about 16.78%. Notably, we found that the levels of influence vaccine-related knowledge and attitudes were related to the behavior of the influenza vaccine.

Spearman's correlation analysis illustrated that there were positive correlations between the influence vaccine-related KAP among HCWs in Chongqing, China. This result supported the KAP theory about the causal chain of KAP (9). Health education on influenza vaccines may be an effective strategy to improve HCWs' KAP related to influenza vaccines (19).

The structural equation model was constructed based on the KAP theory in our study. The KAP theory was developed as a human health promotion model, and it claimed that the change in human behavior could be divided into three continuous processes: knowledge acquisition, belief generation, and practice/behavior formation (20). KAP should have a positive relationship according to KAP theory (21). In our study, the final model showed that there was a significant positive relationship between influenza vaccine-related knowledge and attitudes, knowledge and practice, and attitudes and practice. This research showed that influenza vaccine-related knowledge exhibited a direct relationship with practice and exhibited an indirect effect on behavior through attitude, which indicated that attitudes had a mediating effect between knowledge and behavior. This finding was supported by scholars in other fields, and they also confirmed that knowledge can indirectly affect practice through attitudes (21, 22).

It was worth noting that our study demonstrated that the influenza vaccine-related knowledge of medical staff not only has a direct effect on attitude and behavior, but also indirectly affects behavior through attitude. Unexpectedly, the normalization coefficient index of indirect influence of knowledge on behavior through attitude (0.144) is about two times that of the direct influence on behavior (0.071). This indicates that influenza vaccine-related knowledge has a stronger mediating effect than a direct effect on behavior through influencing attitude, which has not been found in previous studies.

The path coefficient for the direct effect of knowledge on practice was estimated to be β = 0.071, while the indirect effect of knowledge on behavior was 0.144, while the direct effect of knowledge on attitudes was about 0.175. These findings suggested that the influence of knowledge on attitudes and practice was limited. However, the direct effect of attitude on practice was estimated to be β = 0.818. This coefficient implied attitude that has a strong influence on behavior, and also demonstrated that attitude plays an important role in the causal chain of knowledge, attitudes, and behavior.

### Strength and Limitation

As far as we are concerned, this study is the first one to explore the relationship between influenza vaccine-related knowledge, attitudes and behavior among HWCs in Chongqing, China by using SEM. Besides, this research is of great significance for the government to further explore and decide whether to implement the policy of compulsory influenza vaccination targeted at HCWs. However, this study has some limitations. First, there may be a few self-reported HCWs who falsely responded to get a vaccination against influenza due to social pressure, which might cause inevitable bias and lead respondents to provide socially acceptable answers. Second, this research designed to establish the SEM to explore the relationship between knowledge, attitudes, and behavior, but the use of SEM is the inability to explore the inferential causality in this cross-sectional study. Third, we only use the influenza vaccine-related KAP as latent variables according to SEM analysis. However, there are other relevant variables that might not be considered, such as individual characteristics (gender, grade, and major), environmental contexts (family economic annual income and respondents' education level), and social influences (policies and regulations of government) that Wilson and Cleary had put forward (23). Finally, since 2020, the COVID-19 pandemic has spread a lot of influenza-related information, especially HCWs, who will have more opportunities to learn about influenza than before, and they will have a richer knowledge and more positive attitude toward influenza and its vaccines. Our research was conducted before the COVID-19 pandemic, and the results may be quite different from the status quo. Further, more studies on a larger scale are needed in the future.

## Conclusion

The result of this study illustrated that influenza vaccine-related KAP were satisfactory among HCWs, while their willingness to obtain the influenza vaccine shot was not optimistic. According to the SEM, a direct positive relationship was established between influenza vaccine-related knowledge and attitudes, as well as between knowledge and practice and attitudes and practice. The standardized indirect influence of knowledge on behavior through attitude is about two times that of its direct influence on behavior, which indicated that attitude plays a strong mediating role between knowledge and behavior. Our finding supported the causal chain of KAP in the KAP theory. The relationship between potential variables was also found.

## Data Availability Statement

The original contributions presented in the study are included in the article/Supplementary Material, further inquiries can be directed to the corresponding author.

## Ethics Statement

Written informed consent was obtained from the individual(s) for the publication of any potentially identifiable images or data included in this article.

## Author Contributions

All authors listed have made a substantial, direct, and intellectual contribution to the work and approved it for publication.

## Conflict of Interest

The authors declare that the research was conducted in the absence of any commercial or financial relationships that could be construed as a potential conflict of interest.

## Publisher's Note

All claims expressed in this article are solely those of the authors and do not necessarily represent those of their affiliated organizations, or those of the publisher, the editors and the reviewers. Any product that may be evaluated in this article, or claim that may be made by its manufacturer, is not guaranteed or endorsed by the publisher.
